# A DNA barcoding framework for taxonomic verification in the Darwin Tree of Life Project

**DOI:** 10.12688/wellcomeopenres.21143.1

**Published:** 2024-06-24

**Authors:** Alex D. Twyford, Jordan Beasley, Ian Barnes, Heather Allen, Freja Azzopardi, David Bell, Mark L. Blaxter, Gavin Broad, Lucia Campos-Dominguez, Darren Choonea, Liam Crowley, Piotr Cuber, Michael Cunliffe, Alexandra Dombrowski, Brian Douglas, Laura L. Forrest, Ester Gaya, Clementine Greeves, Claire Griffin, Joanna Harley, Michelle L. Hart, Peter W.H. Holland, Peter M. Hollingsworth, Inez Januszczak, Amanda Jones, Paul Kersey, Estelle Kilias, Mara K.N. Lawniczak, Owen T. Lewis, Sahr Mian, Alice Minotto, Raju Misra, Peter O. Mulhair, Lyndall Pereira da Conceicoa, Ben W. Price, Silvia Salatino, Felix Shaw, Olga Sivell, Laura Sivess, Rebekka Uhl, Kieran Woof, Darwin Tree of Life Consortium

**Affiliations:** 1 Institute of Ecology and Evolution, The University of Edinburgh, Edinburgh, Scotland, EH9 3FL, UK; 2 Royal Botanic Garden Edinburgh, Edinburgh, Scotland, EH3 5LR, UK; 3 Natural History Museum, London, England, SW7 5BD, UK; 4 The Marine Biological Association, Plymouth, England, PL1 2PB, UK; 5 Tree of Life, Wellcome Sanger Institute, Hinxton, England, CB10 1SA, UK; 6 Centre for Research in Agricultural Genomics, Barcelona, Spain; 7 Department of Biology, University of Oxford, Oxford, England, OX1 3SZ, UK; 8 Royal Botanic Gardens Kew, Richmond, England, TW9 3AB, UK; 9 Earlham Institute, Norwich, NR4 7UZ, UK

**Keywords:** DNA barcoding, species identification, taxonomy, Britain and Ireland, biodiversity

## Abstract

Biodiversity genomics research requires reliable organismal identification, which can be difficult based on morphology alone. DNA-based identification using DNA barcoding can provide confirmation of species identity and resolve taxonomic issues but is rarely used in studies generating reference genomes. Here, we describe the development and implementation of DNA barcoding for the Darwin Tree of Life Project (DToL), which aims to sequence and assemble high quality reference genomes for all eukaryotic species in Britain and Ireland. We present a standardised framework for DNA barcode sequencing and data interpretation that is then adapted for diverse organismal groups. DNA barcoding data from over 12,000 DToL specimens has identified up to 20% of samples requiring additional verification, with 2% of seed plants and 3.5% of animal specimens subsequently having their names changed. We also make recommendations for future developments using new sequencing approaches and streamlined bioinformatic approaches.

## Plain language summary

Identifying species based solely on their morphology can be difficult. DNA-based identification using DNA barcoding can aid species identification, but can be challenging to implement in biodiversity projects sampling diverse organismal groups. Here, we describe the development and implementation of DNA barcoding for the Darwin Tree of Life Project (DToL), which aims to sequence and assemble high quality reference genomes for all eukaryotic species in Britain and Ireland. We discuss how a standardised approach has been adapted by each partner to suit different organismal groups, show the efficacy of this approach for confirming species identities and resolving taxonomic issues, and make recommendations for future developments.

## Disclaimer

The views expressed in this article are those of the authors. Publication in Wellcome Open Research does not imply endorsement by Wellcome.

Species identification is central to biodiversity research but is hampered by a multitude of challenges, from biological issues such as cryptic diversity that prevent the unambiguous assignment of names in complex species groups, to technical issues associated with degraded or incomplete specimens ( Padial *et al.*, 2010). Species identification becomes ever more important in the era of biodiversity genomics, where a large investment is made to collect, sequence and assemble complete genomes, and where mistaken identification could cause pervasive issues downstream. DNA barcoding - the sequencing of standard DNA regions to differentiate species ( Hebert *et al.*, 2003) - is one approach that may provide a valuable independent confirmation of species identity in large biodiversity genomic projects ( Lawniczak *et al.*, 2022a). DNA barcoding has been widely used to inform species identification and for estimating species diversity in a wide range of studies, ranging from ecological forensics, vegetation surveys, community phylogenetics and environmental monitoring ( DeSalle & Goldstein, 2019; Gostel & Kress, 2022; Kress *et al.*, 2015), but is not routinely implemented as an identification tool in most studies generating complete genomes. However, DNA barcoding has great promise for rapidly confirming species identities before a sample enters the genome sequencing pipeline.

Here, we develop a framework for DNA barcoding for the Darwin Tree of Life (DToL) project, and consider how this approach may be adopted by other large-scale biodiversity genome initiatives. DToL has the aim of sequencing and assembling high quality reference genomes for all eukaryotic species present in Britain and Ireland ( Darwin Tree of Life Project Consortium, 2022). As a large-scale project sampling the full regional diversity of eukaryotic life, DToL faces many potential issues with species identification. Initially, all specimens are identified in the field at the point of collection by taxonomic experts. In most cases, the initial taxonomic identification will be correct. However, taxonomic complexes and cryptic species in particular pose problems, where species misidentification or uncertainty must be anticipated ( Bickford *et al.*, 2007). This challenge is especially acute in certain groups of organisms. This includes fungi, where morphological characters alone often fail to tell species apart ( Lücking *et al*., 2020), as well as many arthropods, where identification is often impossible in the field as it requires examination of internal or concealed structures. The original species identifications are therefore verified by DNA barcoding using taxon appropriate loci (mitochondrial, plastid or ribosomal RNA). Moreover, despite centuries of intensive study of the natural history of Britain and Ireland ( Allen, 1976; Harding, 1992; Pocock *et al.,* 2015; Stroh *et al.,* 2023), the integration of new morphological observations with DNA barcoding and genomic sequencing will inevitably lead to species discovery and taxonomic change. This is particularly pertinent as new species colonise Britain and Ireland due to range expansion driven by factors such as anthropogenic introductions and changes in climate ( Martay *et al.*, 2017). Finally, the scale of sampling and the extensive set of downstream laboratory processes in DToL and other large biodiversity genomic initiatives make the potential for sample tracking an important benefit of generating barcodes prior to sequence submission. Here, a comparison between the DNA barcode generated after specimen collection, and the barcode sequence recovered from the genome assembly, can provide a valuable check for such mistakes ( Darwin Tree of Life Project Consortium, 2022).

## Current status of UK barcoding reference databases

DNA barcoding for DToL is aided by the extensive DNA barcoding reference datasets that have been generated for diverse British and Irish species, although there are notable limitations of the currently available data. Natural England commissioned a comprehensive assessment of the DNA sequences available in the major DNA reference libraries for approximately 76,000 eukaryotic species in the UK. The results of this formal gap analysis revealed that 52% of UK species had some publicly accessible DNA sequence data ( Price *et al.*, 2020), varying widely across different databases (e.g. the Barcoding of Life Data System, BOLD; Genbank; European Nucleotide Archive, ENA). Within BOLD, for example, at least one barcode sequence was available for 42.5% of UK species, however in many cases this was not from a specimen collected from the UK. Strikingly, imposing stringent quality standards on the barcoding data accessible in BOLD resulted in a reduction of UK species coverage to just 4%. Although around half of UK species are represented in reference databases such as BOLD, that figure is significantly taxonomically biased toward, for example, invasive species, or those with a conservation status ( Price *et al.*, 2020). Unsurprisingly many species that are challenging to identify (e.g. species that are small or show cryptic species differences), or rarely encountered, are poorly represented in reference libraries, or may be misidentified. DNA sequence quality, metadata completeness, and the accuracy of species identification varied markedly both within and between reference libraries. The paucity of funding, expertise, and capacity across various domains, including taxonomy, molecular laboratory techniques, bioinformatics, as well as quality control and assurance have been identified as the main challenges in building up more accurate and complete barcoding reference libraries. To this end, DToL offers a unique opportunity to leverage its expertise in these areas to expand the coverage of UK species with high-quality, rigorously verified data.

## Embedding DNA barcoding in the sample workflow

The DToL process starts with the collection of samples by specialist collectors at ‘Genome Acquisition Laboratories’ (GALs) from diverse locations across Britain and Ireland, including field sites and from living collections. A specimen is identified to species level at the point of collection, or soon after in the laboratory, based on morphological characteristics by a taxonomic expert. This taxon identification is stored with other key metadata in a standardised sample manifest ( Lawniczak *et al.*, 2022b). Where possible, tissue for DNA barcoding is taken from the specimen that is used for genomic sequencing. For some taxa, additional specimens are collected as morphological and DNA barcode vouchers and to provide additional material for sequencing if required at a later date. In other cases, small samples or organisms need to be cultured to bulk up enough biomass (e.g., some fungi, protists), increasing the amount of material that requires DNA barcoding to ensure unwanted contaminants are not genome sequenced.

No single set of DNA barcoding procedures would be suitable for the diversity of organisms processed by DToL. For example, the standard processing procedure for adult terrestrial arthropods involves the removal of one (or more) legs which are preserved in 70% ethanol prior to amplification of the CO1 region ( Crowley *et al.*, 2023; Pereira *et al.*, 2022), whereas for vascular plants above-ground tissue is stored in desiccating silica gel prior to amplification of rbcL and ITS2 (or rbcL and trnL-F for ferns). In fungi, tissue or spores are plated in petri dishes for in vitro culturing and subsequently identified with ITS. After sequence generation, the process of interpreting the data differs depending on the preferred search query approach, the extent of the reference database, and the ability of a given locus to tell species apart. Despite differences in the interpretation process, a general set of rules are necessary across organismal groups to ensure consistent working practices and to allow DNA barcoding to operate efficiently at scale. As such, the development of DToL DNA barcoding aimed to:

1. Establish a network of DToL DNA barcoding hubs that can process and generate sequences for diverse samples at scale.
2. Put in place a flexible bioinformatic workflow that can be tailored to the analysis and interpretation of diverse samples.
3. Establish principles for data sharing and publication that allow rapid dissemination of data in a fair and equitable manner.

**Figure 1.**
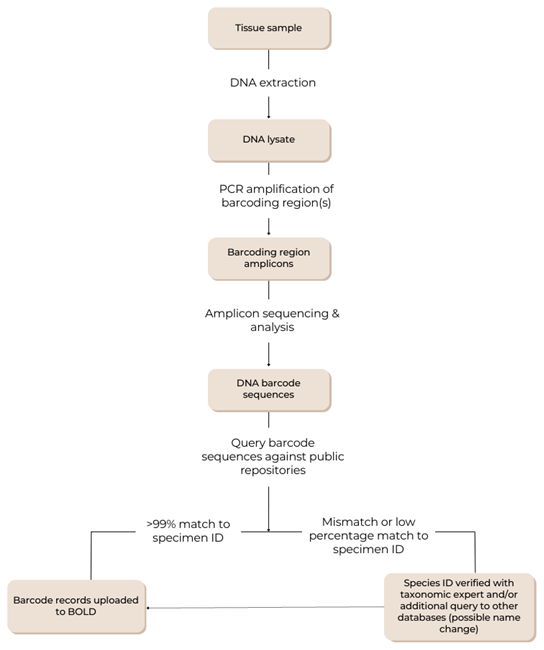
General DNA barcoding workflow for DToL. Specific lab protocols, barcoding regions and query parameters vary between taxa and barcoding hubs.

Our development is focused around the strategy outlined in Figure 1, where a general and standardised set of practices are adapted to: (1) organism specific tissue processing and DNA extraction needs, including variation in specimen size, tissue type, and tissue preservation method, (2) PCR amplification requirements and consideration of the most suitable primers, (3) the best suited bioinformatic approach and reference database.

Finally, as there are a small number of taxa where morphological identification is certain, but barcode sequencing is known to have very low success, we have instituted a barcoding exemption list. This allows samples from these small number of groups to be submitted without barcoding being attempted. The list is curated by the DToL taxon working groups, and kept under review as new approaches may enable currently intractable groups to be barcode sequenced (discussed more, below).

## Establishing a network of DNA barcoding hubs

To establish protocols and deliver rapid DNA barcoding across a wide phylogenetic diversity of samples, we established a network of four core DNA barcoding hubs, each with a taxonomic focus ( Table 1). These core DNA barcoding hubs work with four satellite hubs and analysis partners providing specific additional expertise. Each hub is tasked with adapting the core DNA barcoding workflow ( Figure 1) for their nominated taxon groups, to ensure suitable data standards and sequencing success rates given the specific challenges of a given taxon group. A guiding principle is that for DNA barcoding to be delivered at scale, it must follow clearly defined standard operating procedures defined at a coarse taxonomic level, rather than requiring species-specific optimisation and manual intervention wherever possible. Full protocols for each hub are available here: https://protocols.io/view/dnabarcoding-sops-for-thedarwin-tree-of-life-proc4yeyxte.

**Table 1.** Roles of barcoding hubs in DToL.

Partner	Role in DNA barcoding
*Core barcoding hubs*	
Marine Biological Association (MBA)	Marine organisms
Natural History Museum (NHM)	Animals from NHM and Wytham Woods collections, jointly process fungi with RBG Kew and marine organisms with MBA
Royal Botanic Garden Edinburgh (RBGE)	Land plants, lichens
Protist Group, University of Oxford	Protists
*Satellite hubs and analysis partners*	
Royal Botanic Gardens Kew (RBG Kew)	Jointly process fungi, with NHM
Wytham Genome Project, University of Oxford	Interpretation of data from Wytham Woods collections, sequenced at NHM
University of Edinburgh	Support RBGE in the analysis of vascular plant samples
Sanger Institute	DNA barcoding of local collections not handled by other GALS

Each hub receives DNA barcoding tissue samples or PCR amplicons from the GALs, along with sample metadata, ready for processing. These samples are usually small and shipped at room temperature as there are relatively low input quantity and quality requirements for barcoding. All hubs perform their own DNA extractions tailored to the broad organismal group, as well as PCR reactions with one of the current catalogue of taxon-specific primer sets ( Table 2).

**Table 2.** Primer details for each DNA barcoding locus. Table includes: the Genome Acquisition Lab (GAL) or Barcoding hub responsible for a given taxon group; taxon group with defined sets of DNA barcoding loci; DNA barcoding database used for search queries (multiple databases searched for some groups); forwards and reverse primers separated by a slash; ENA marker sequence checklist classification for depositing the data on ENA; primer sequences; locus description and notes providing detailed information and typical amplicon size.

GAL or Barcoding hub	Taxon group	DNA barcoding loci	DNA barcoding database	Primers	ENA marker sequence checklist	Forward Primer		Reverse Primer		Locus description and notes
						Name	Sequence	Name	Sequence	
RBG Edinburgh	Seed plants	ITS2	BOLD/NCBI	ITS_S2F/ITS_S3R	ITS rDNA	ITS_S2F	ATGCGATACTTGGTGTGAAT	ITS_S3R	GACGCTTCTCCAGACTACAAT	Nuclear internal transcribed spacer 2, flanked by partial 5.8S and 26S
		rbcL	BOLD/NCBI	rbcLa-F/ rbcLajf634R;	Single CDS genomic DNA	rbcLa-F	ATGTCACCACAAACAGAGACTAAAG	rbcLajf634R	GAAACGGTCTCTCCAACGCAT	Partial sequence (607bp) of plastid- encoded rubisco gene
	Ferns, Lycophytes	rbcL	BOLD/NCBI	rbcLa-F/ rbcLajf634R	Single CDS genomic DNA	rbcLa-F	ATGTCACCACAAACAGAGACTAAAG	rbcLajf634R	GAAACGGTCTCTCCAACGCAT	Partial sequence (607bp) of plastid- encoded rubisco gene
		trnL-trnF	BOLD/NCBI	trnL.C_bryo/ trnFGAAf	Multi- Locus Marker	trnL.C_bryo	CGAAATTGGTAGACGCTGCG	trnFGAAf	ATTTGAACTGGTGACACGAG	Partial sequence of the first exon of the plastid trnL gene, the intragenic spacer, the second exon of the trnL gene, the trnL-trnF intergenic spacer, and partial sequence of the trnF gene
	Liverworts, Mosses, Hornworts	ITS2	BOLD/NCBI	ITS2.seqF/ ITS.4bryo	ITS rDNA	ITS2.seqF	AACAACTCTCAGCAACGG	ITS.4bryo	TCCTCCGCTTAGTGATATGC	Nuclear internal transcribed spacer 2, flanked by partial 5.8S and 26S
		rbcL	BOLD/NCBI	rbcLa-F/ rbcLajf634R	Single CDS genomic DNA	rbcLa-F	ATGTCACCACAAACAGAGACTAAAG	rbcLajf634R	GAAACGGTCTCTCCAACGCAT	Partial sequence (607bp) of plastid- encoded rubisco gene
		psbA-trnH	BOLD/NCBI	psbA501F/trnHR	Multi- Locus Marker	psbA501F	TTTCTCAGACGGTATGCC	trnHR	GAACGACGGGAATTGAAC	c. 500 bases of plastid-encoded photosystem gene, short intergenic spacer and partial trnH gene
	Lichen- forming fungi	ITS	BOLD/ NCBI/ UNITE	ITS1-F/ITS4	ITS rDNA	ITS1-F	CTTGGTCATTTAGAGGAAGTAA	ITS4	TCCTCCGCTTATTGATATGC	Internal transcribed spacer 1, 5.8S and internal transcribed spacer 2, flanked by partial 18S and 26S
		nuLSU	BOLD/ NCBI/ UNITE	LROR/LR5	rRNA Gene	LROR	ACCCGCTGAACTTAAGC	LR5	ATCCTGAGGGAAACTTC	Nuclear large subunit ribosomal RNA, partial sequence
		mtSSU	BOLD/ NCBI/ UNITE	mrSSU1/ mrSSU2R	rRNA Gene	mrSSU1	AGCAGTGAGGAATATTGGTC	mrSSU2R	CCTTCGTCCTTCAACGTCAG	Mitochondrial small subunit rDNA, partial sequence; trialling a different reverse primer (mtSSU3R) for a slightly longer fragment
NHM	Arthropoda, Mollusca	COI	BOLD/NCBI	Primer cocktail: LCO1490 + LepF/HCO2198 + LepR	COI gene	LCO1490	GGTCAACAAATCATAAAGATATTGG	HCO2198	TAAACTTCAGGGTGACCAAAAA ATCA	Partial sequence (c. 710bp) of COI gene
						Lep_F1	ATTCAACCAATCATAAAGATATTGG	Lep_R1	TAAACTTCTGGATGTCCAAAAA ATCA	Partial sequence (c.648bp) of COI gene
	Gastropods	16S	BOLD/NCBI	16SAR_gpd_ S/16SBR_gpd_S	rRNA Gene	16SAR_gpd_S	CGCCTGTTTAWCAAAAACAT	16SBR_gpd_S	CCGGTYTGAACTCAGATCAGAT CAYGT	Partial sequence (c. 570 bp) of 16S rRNA gene
	Vertebrates	CO1	BOLD/NCBI	C_VF1LFt1 – C_VR1LRt1 (Mammal cocktail); LepF1_t1, VF1_t1, VF1d_t1, VF1i_t1, LepR1_t1, VR1d_t1, VR1_t1, VR1i_t1	COI gene	LepF1_t1	TGTAAAACGACGGCCAGTAT TCAAC CAATCATAAAGATATTGG	LepR1_t1	CAGGAAACAGCTATGACTAAAC TTC TGGATGTCCAAAAAATCA	Partial sequence (c. 700bp) of COI gene
						VF1_t1	TGTAAAACGACGGCCAGTTCT CAAC CAACCACAAAGACATTGG	VR1d_t1	CAGGAAACAGCTATGACTAGAC TTC TGGGTGGCCRAARAAYCA	Partial sequence (c. 700bp) of COI gene
						VF1d_t1	TGTAAAACGACGGCCAGTTCT CAAC CAACCACAARGAYATYGG	VR1_t1	CAGGAAACAGCTATGACTAGAC TTC TGGGTGGCCAAAGAATCA	Partial sequence (c. 700bp) of COI gene
						VF1i_t1	TGTAAAACGACGGCCAGTTCT CAAC CAACCAIAAIGAIATIGG	VR1i_t1	CAGGAAACAGCTATGACTAGAC TTC TGGGTGICCIAAIAAICA	Partial sequence (c. 700bp) of COI gene
Oxford	Amoebozoa, Alveolata, Cercozoa, Chlorophyta, Cryptophyta, Euglenozoa	18S (V4,V9)	SILVA/PR2/ NCBI	TAReuk454FWD1/ TAReukREV3, E572F/E1009R, 1389F/1510R, 1391F/EUKBr	rRNA Gene	TAReuk454FWD1	CCAGCASCYGCGGTAATTCC	TAReukREV3	ACTTTCGTTCTTGATYRA	Partial sequence (c.417 bp) of 18s sRNAgene (V4)
						E572F	CYGCGGTAATTCCAGCTC	E1009R	CRAAGAYGATYAGATACCRT	Partial sequence (c.440bp) of 18s sRNAgene (V4)
						1389F	TTGTACACACCGCCC	1510R	CCTTCYGCAGGTTCACCTAC	Partial sequence (c.170 bp) of 18s sRNAgene (V9)
						1391F	GTACACACCGCCCGTC	EUKBr	TGATCCTTCTGCAGG TTCACCTAC	Partial sequence (c.170 bp) of 18s sRNAgene (V9)
	Ochrophyta	18S (V4,V9)	SILVA/PR2/ NCBI	TAReuk454FWD1/ TAReukREV3, E572F/E1009R, D512/D978rev, 1389F/1510R, 1391F/EUKBr	rRNA Gene	TAReuk454FWD1	CCAGCASCYGCGGTAATTCC	TAReukREV3	ACTTTCGTTCTTGATYRA	Partial sequence (c.417 bp) of 18s sRNAgene (V4)
						E572F	CYGCGGTAATTCCAGCTC	E1009R	CRAAGAYGATYAGATACCRT	Partial sequence (c.440bp) of 18s sRNAgene (V4)
						D512	TTGTACACACCGCCC	D978rev	GACTACGATGGTATCTAATC	Partial sequence (c. 400bp) of 18s sRNAgene (V4)
						1389F	TTGTACACACCGCCC	1510R	CCTTCYGCAGGTTCACCTAC	Partial sequence (c.170 bp) of 18s sRNAgene (V9)
						1391F	GTACACACCGCCCGTC	EUKBr	TGATCCTTCTGCAGGTTCACCT AC	Partial sequence (c.170 bp) of 18s sRNAgene (V9)
RBG Kew	Fungi	ITS	NCBI/ UNITE	ITS1F/ITS4	ITS rDNA	ITS1F	CTTGGTCATTTAGAGGAAGTAA	ITS4	TCCTCCGCTTATTGATATGC	Internal transcribed spacer 1, 5.8S and internal transcribed spacer 2 (c. 500bp)
MBA	Phaeophytes	COI	BOLD/NCBI	GazF2/GazR2	COI gene	GazF2	CCAACCAYAAAGATATWGGTAC	GazR2	GGATGACCAAARAACCAAAA	Partial sequence (c. 700 bp) michondrial cytochrome oxidase I gene 5P end
	Phaeophytes, Rhodophytes	COI	BOLD/NCBI	GWSFn/GWSRx	COI gene	GWSFn	TCAACAAAYCAYAAAGATATYGG	GWSRx	ACTTCTGGRTGICCRAARAAYCA	Partial sequence (c. 700 bp) michondrial cytochrome oxidase I gene 5P end
		LSU D2/D3	BOLD/NCBI	T16N/T24U	rRNA Gene	T16N	AMAAGTACCRYGAGGGAAAG	T24U	SCWCTAATCATTCGCTTTACC	D2/D3 region partial sequence (c. 200 bp) of large subunit ribosomal DNA gene
	Rhodophytes	rbcL	BOLD/NCBI	rbcLa-F/rbcLa-R	Single CDS genomic DNA	rbcLa-F	ATGTCACCACAAACAGAGACTAAAGC	rbcLa-R	GTAAAATCAAGTCCACCRCG	Partial sequence (c. 500bp) of plastid-encoded rubisco gene
		rbcL	BOLD/NCBI	F57/rbcLrevNEW	Single CDS genomic DNA	F57	GTAATTCCATATGCTAAAATGGG	rbcLrevNEW	ACATTTGCTGTTGGAGTYTC	Plastid enclosed rubisco large subunit gene (c. 1,300 bp)
		rbcL	BOLD/NCBI	F57/TLR1	Single CDS genomic DNA	F57	GTAATTCCATATGCTAAAATGGG	TLR1	AAYTCWGCTCTTTCRTAYAT	Plastid enclosed rubisco large subunit gene (c. 650 bp) partial 5P end
		rbcL	BOLD/NCBI	F57/TLR4	Single CDS genomic DNA	F57	GTAATTCCATATGCTAAAATGGG	TLR4	AAYTCWGCCCTTTCRTACAT	Plastid enclosed rubisco large subunit gene (c. 650 bp) partial 5P end
		rbcL	BOLD/NCBI	AcroF1/ rbcLrevNEW	Single CDS genomic DNA	AcroF1	AGCTCAAGCCGCAGCAGGAG	rbcLrevNEW	ACATTTGCTGTTGGAGTYTC	Plastid enclosed rubisco large subunit gene (c. 650 bp) partial 3P end
		rbcL	BOLD/NCBI	TLF1/rbcLrevNEW	Single CDS genomic DNA	TLF1	TCYCARCCWTTYATGCGCTGG	rbcLrevNEW	ACATTTGCTGTTGGAGTYTC	Plastid enclosed rubisco large subunit gene (c. 650 bp) partial 3P end
		rbcL	BOLD/NCBI	M13LF3/M13RX	Single CDS genomic DNA	M13LF3	TGTAAAACGACGGCCAGTACH AAYCAYAARGATATHGG	M13RX	CAGGAAACAGCTATGACACTTC TGGRTGICCRAARAAYCA	Plastid enclosed rubisco large subunit gene (c. 1,300 bp)
	Chlorophyte macroalgae	tufA	BOLD/NCBI	tufGF4/tufAR	Single CDS genomic DNA	tufGF4	GGNGCNGCNCAAATGGAYGG	tufAR	CCTTCNCGAATMGCRAAWCGC	Partial sequence elongation factor Tu (c. 600 bp)
		rbcL	BOLD/NCBI	GrbcLFi/1385R	Single CDS genomic DNA	GrbcLFi	TCTCARCCWTTYATGCGTTGG	1385R	AATTCAAATTTAATTTCTTTCC	Plastid enclosed rubisco large subunit gene (c. 650 bp) partial 3P end
	Alismatales (seagrasses)	ITS	BOLD/NCBI	P674/P675	ITS rDNA	P674	CCTTATCATTTAGAGGAAGGAG	P675	TCCTCCGCTTATTGATATGC	Internal transcribed spacer 1, 5.8S and internal transcribed spacer 2 (c. 700bp)
	Cryptophytes	psaA	SILVA/PR2/ NCBI	psaA.F529/ psaA.1748R	Single CDS genomic DNA	psaA.F529	GGWTGGTTYCAYTAYCAYAARKCWGC	psaA.1748R	CCCAWGCHGAWMYTTGRCAW GTWCC	Partial sequence photosystem I P700 chlor-ophyll a apoprotein A1 (c. 1050 bp)
	Protists	18S V4	SILVA/PR2/ NCBI	E572F/E1009R	rRNA Gene	E572F	CYGCGGTAATTCCAGCTC	E1009R	AYGGTATCTRATCRTCTTYG	Partial sequence 18S small subunit V4 region (c.450 bp)
		18S V9	SILVA/PR2/ NCBI	1389F/1510R	rRNA Gene	1389F	TTGTACACACCGCCC	1510R	CCTTCYGCAGGTTCACCTAC	Partial sequence 18S small subunit V4 region (c.150 bp)
	Marine fungi and lichens	ITS	NCBI/ Species Fungorum/ UNITE	ITS1F/ITS4	ITS rDNA	ITS1F	CTTGGCATTTAGAGGAAGTAA	ITS4	TCCTCCGCTTATTGATATGC	Internal transcribed spacer 1, 5.8S and internal transcribed spacer 2 (c. 500bp)
		18S	NCBI/ Species Fungorum/ UNITE	NS1/FR1	rRNA Gene	NS1	GTAGTCATATGCTTGTCTC	FR1	AICCATTCAATCGGTAIT	Partial sequence 18S small subunit V2/V9 region (c.1650 bp)

Sequencing is subsequently performed using one of two approaches. Sanger sequencing has been adopted at most hubs as it enables rapid turnaround and is flexible for managing small and variable batch sizes encountered with field-collections. More recently, the NHM is in the process of transitioning to Oxford Nanopore Technologies (ONT), with ~250 samples uniquely indexed and multiplexed in a single library, which is sequenced using the Flongle adaptor for the ONT MinION. This approach is especially useful when dealing with large numbers of samples that use the same primers, with added benefits including information gained in samples with a mix of organisms. There are also cost benefits, with this implementation of ONT with suitably high multiplexing costing a third of Sanger sequencing (£2.23 vs £6.60 per sample), in line with the costings previously described by Cuber *et al.* (2023). RBGE and other hubs are also testing the efficacy and scalability of ONT. Following data production common data standards have been adopted by all hubs to ensure consistency and quality. For PCR amplicons that are Sanger sequenced this means having both directions sequenced as standard, with low quality bases trimmed ( Hanner, 2012), while for ONT this means filtering low quality bases.

## Analysing diverse DNA barcoding data

Following sequence data production and data quality control, sequences move to bioinformatic analysis. The bioinformatics workflow for DToL DNA barcoding varies between barcoding hubs, and by technology. For example, the NHM Sanger sequencing pipeline starts with processing of the sequencing data (AB1 trace files) with an in-house Nextflow analysis pipeline, including trimming of adapters and poor quality bases with Trimmomatic, and forward and reverse strand merging with Pipebar. Subsequently BOLD and NCBI database queries are made with bold_identification and Blastn ( Cuber *et al.*, 2023). In contrast, ONT sequencing data are processed using the ONTbarcoder software, followed by database queries using BOLDigger.

The approach used for interpreting sequence queries creates numerous challenges for DNA barcoding. The efficacy of DNA barcoding for confirming species identification is taxon specific and depends on biological attributes of a taxon group as well as the availability of key contextual information. Firstly, there are intrinsic differences in the efficacy of DNA barcoding for identification in different organismal groups. Groups where DNA barcode sequences track species boundaries can be interpreted with greater confidence than groups where DNA barcodes offer poor species discrimination (such as where hybridisation is rampant and/or where speciation is recent, or where the barcode regions show lower levels of sequence divergence, Hollingsworth *et al.*, 2011). Secondly, query resolution is linked to the completeness of the DNA barcode reference library, with different interpretation required for groups with near-complete taxon coverage in the reference database, as opposed to those with extensive missing data.

Given the complex nature of species differences and the differing availability of reference data, DToL has adopted a suite of bioinformatics approaches that are chosen for different taxonomic groups. These are:

a. The ‘closest matches’ approach. A sequence search using BOLD, GenBank, SILVA, UNITE or other database, followed by looking at the closest matches in the results table.
b. Use of the Barcode Index Number (BIN) system ( Ratnasingham & Hebert, 2013). A sequence search is made in BOLD, where search sequences falling within a BIN threshold are considered consistent matches. Only currently relevant for animals using the CO1 barcode region.
c. The ‘user specified threshold’ approach. A sequence search, where matches below a group-specific percentage divergence threshold are treated as consistent matches. This approach is most relevant for groups with patchy taxon representation in the database.
d. Phylogenetic approach. A phylogenetic tree that places the search sequence either with the same species in the reference dataset, or with the same genus, is considered a definitive match or a consistent match, respectively.

Based on these analyses, each query falls into a different match type, summarised in Table 3.

**Table 3.** Description of the possible match categories and outcomes from DNA barcoding queries against reference libraries.

Match type	Description	Outcome
Definitive match	Query sequence has an exact match to a sequence annotated with the same taxon name or a nomenclatural synonym in the reference database. Dataset includes comprehensive sampling of all relevant congeneric taxa.	Sample continues to genome sequencing.
Consistent match	Query sequence matches a sample in the reference database in a manner consistent with the correct taxon identification. This may be: *(a)* a match to a suite of species which includes the target taxon and other related taxa (i.e. lack of resolution); *(b)* a match to the right genus but no match to the target species due to it not being present in the reference database (lack of reference data).	Although a definitive match is not achieved, this is often expected, and there is no indication from the barcode data that the sample has been misidentified. Sample continues to genome sequencing by default, or may be subject to sample verification if there are outstanding taxonomic questions in the group.
Inconsistent match	Query sequence match suggests an issue with the identification of the specimen or issue with the reference dataset. This may manifest as: *(a)* a match to a different but closely related species where there is a well populated and highly resolved reference library, indicating that a congeneric species has been mistakenly sampled (misidentification), or that there is previously undetected intra- specific variation in the barcode region (polymorphism); *(b)* a match to another distantly related species, genus or family even though the focal taxon is included in the database. This may be due to the wrong name being applied to a specimen (misidentification), a sample handling error (sample switch), potential contamination, or an identification error in the reference database.	Sample verification required.
No match	Query sequence returns no near match, and no congeneric representatives are present in the database, therefore there is no relevant contextual information available to interpret the results. This only applies to certain organismal groups with limited data availability, discussed below.	Sample continues to genome sequencing (in some cases, following sequencing additional samples or morphological reverification).

Samples with a definitive match proceed to shipping and genome sequencing. The default outcome for samples with a consistent match or no match is to proceed to shipping, however, individual barcoding hubs may choose to further investigate some specimens, for example if: (1) the taxonomic expert wants to revisit the specimen name in light of the barcode data, (2) if a ‘no match’ result is returned, when some level of match may be expected. Samples with an inconsistent match, on the other hand, have a default outcome of manual intervention and sample verification. The specific actions will vary, but will typically include checking for sample switches in the lab, repeating DNA barcoding, checking unmerged forward and reverse reads, and/or checking the name associated with the specimen. In some cases, the interpretation of DNA barcode data can be more challenging, and a simple search within a public library or repository is not sufficient. It often requires systematic expertise and the placement of that DNA barcode in a phylogenetic context for an accurate ID, a task only possible when taxonomic experts are at hand, such as in DToL.

Each of these outcomes, above, are determined by the barcoding hub (or analysis partner) for a defined taxonomic group and a specific bioinformatic approach, to ensure samples are handled consistently. For example, in flowering plants where two barcoding loci are used, and where the barcoding loci have different levels of resolution, the interpretation categories can be augmented as follows:

- Definitive match. *At least one locus has a definitive match, and other locus/loci that do not have a definitive match have at least a consistent match (e.g. genus-level match) and this lower level of resolution is as expected for that barcoding locus.*
- Consistent match. *At least one locus has a match to a congeneric sample (the other allowed to be missing data/no match).*
- Inconsistent match. *At least one locus has a clear match to the ‘wrong taxon’.*
- No match. *No sequences from the same genus/family are available for either locus in the reference database.*

## Integrating DNA barcoding into the core DToL bioinformatic workflow

All data are submitted and made fully accessible in public databases once they have passed initial quality control checks of sequence quality and specimen identity. Barcoding data for animals, plants and fungi are deposited in BOLD, providing wider accessibility of the sequence data ( Ratnasingham & Hebert, 2007). BOLD allows the upload of sequences and trace files, along with metadata, that are then queried in the context of millions of available reference sequences. Our approach is for data to be uploaded at the point of production, with specimen names amended as required after sequence searches and evaluation. DNA barcoding hubs are encouraged to maintain sequence data in BOLD within the “Darwin Tree of Life [DTOL]” container and to tag them with ‘DToL’, improving searchability of DToL data. For taxa not supported by BOLD, such as protists, samples are submitted to INSDC.

At present, BOLD supports taxonomic searches for CO1 for animals, rbcL and matK for plants, and ITS for fungi. For protists, where the V4 or V9 region of 18S rRNA gene is sequenced, we have chosen to query the databases at SILVA ( Quast *et al.*, 2013) or PR2 ( Guillou *et al.*, 2012), while for fungi, we additionally use UNITE ( Nilsson *et al.*, 2019) given the reduced amount of fungal sequences in BOLD.

One additional application of the DNA barcoding data is for sample tracking through the genome production pipeline at the Sanger Institute. Currently, the relevant DNA barcode sequences are extracted from the raw Pacific Biosciences (PacBio) High Fidelity (HiFi) reads at the point of production, searched in the relevant database (BOLD in most cases), and the top matches are examined. The expectation is that the top match will be from the same species as the query specimen. These barcode query summaries are reported in the Tree of Life QC interface ( https://tolqc.cog.sanger.ac.uk/). Since manifest version 2.5 (implemented December 2023), BOLD specimen ID has been submitted with other sample metadata by the GALs. In future updates of the data portal, this will allow the specific barcode sequence from the genome specimens to be recovered from BOLD, improving sample tracking. For protists submitted in INSDC that do not have a BOLD ID, DToL sequence identifiers are linked to INSDC sequence identifiers via a simple two column barcode manifest.

## Publication and data dissemination

The individual DNA barcode sequences generated by DToL primarily support taxon verification, with each verified species featuring in its own genome note that reports the genome assembly ( Threlfall & Blaxter, 2021). More generally, a major outcome from DToL DNA barcoding is a large set of standardised sequences associated with a specimen with a validated name provided by a taxonomic expert. This high quality, curated data will form an important reference resource for species in Britain and Ireland. In some cases, these data can be integrated with existing DNA barcode reference libraries (e.g., UKBOL: https://www.ukbol.org/, Barcode UK, Jones *et al.,* 2021), populating sections with no existing data, or supplementing the dataset with additional samples allowing the analysis of diagnostic sequences and polymorphism. For example, Barcode UK is a DNA barcoding resource for 1,482 UK flowering plant and conifer species, and DToL data is providing sequences for additional native and alien taxa not represented in the current barcode library.

## The workflow in practice

DNA barcode data have been generated and interpreted for over 12,000 DToL samples as of December 2023. Here, we consider our experiences from two focal groups, animal samples processed by the NHM, and plant samples processed by RBGE, as well as implications for other taxon groups.

For animals, we have processed over 10,525 samples. The majority of those samples (~85%) were Arthropoda, followed by Annelida (~5%), Mollusca (~3.4%), and Echinodermata (~1.4%). From the remaining samples, there is a notable representation of: Bryozoa, Nemertea, Cnideria, Hemichordata, Cnidaria, Chordata, and Platyhelminthes. The BOLD query produced a match with the expected species in 59.2% of cases, eliminating the need for further checks. Approximately 20% of the samples required additional verification. This could be attributed to various reasons, such as a mismatch between the BOLD hit and the expected species, or when multiple species were matched. In some instances, the barcode was not present in the BOLD database, indicating it was a new entry. Upon verification by taxonomic experts, 3.5% of the samples had their names changed from the identifier ID to the BOLD ID. The remaining samples were failures of various categories: failure to merge the forward and reverse reads for sanger sequence data (~3%), sample contamination (~1.3%), extraction/PCR failure (~2%), BOLD matches were too low to be informative (~0.8%) or unknown/unclassified (~14.1%). The failure rate for non-marine animals is approximately 2%, whereas for marine animals it is significantly higher (~50%) due to the lower success rate of the quick DNA extraction protocol and the use of universal barcoding primers, as well as the higher levels of contaminants and inhibitors present in the samples. Ongoing development work is focused on taxonomic groups with lower success rates, including the utilisation of different extraction protocols and taxon-specific primers.

There have been many cases where DNA barcoding has clinched animal identification, including of species new to Britain. A parasitoid wasp collected in Beinn Eighe was identified in the field as *Plectiscus ridibundus*, a common and widespread species, but the DNA barcode matched to both *P. ridibundus* and *P. callidulus*. The specimen was then compared to collection specimens at the NHM by a taxonomic expert and subsequently renamed as *P. callidulus*, a first record for Britain. Further UK specimens could then be found which had been misidentified. Another example where barcoding aided ichneumonid wasp identification was with a sample collected on Winterton Dunes at a DNA Bioblitz in 2022. Originally, it was identified as *Lissonota lineata*, a rarely found coastal species. The DNA barcode matched Swedish barcode records of *L. confusa* which had been overlooked in much of Europe. It now seems possible that *L. lineata* does not occur in the UK after all. Of course, DNA barcoding can also bring into question morphology-based identifications. Two noctuid moths were collected in Kent which, on wing pattern, looked exactly like *Agrotis catalaunensis*, a southern European species never recorded in mainland Britain. Some experts agreed, based on the photos. However, barcodes suggested they were in fact *Agrotis puta*, a very common British moth, and therefore there is a confusing wing pattern variety.

For plants, we have processed over 1,250 samples, including 869 seed plants, 343 bryophytes and 41 ferns, representing approximately 50% of the British native flora. Our experience suggests DNA extraction, amplification and sequencing can be implemented effectively at scale, with mostly low levels of sample-specific dropout. For example, for 820 of 869 (95%) seed plants we have successfully recovered sequences for both loci used for barcoding. The most significant systematic dropout we have observed is for ferns and lycophytes, where the ITS2 locus proved problematic; based on our low sequence success and conversations with fern taxonomists, we have instead sequenced the plastid trnL-trnF locus, with over 90% of ferns now having sequence data for at least two barcode markers. For the seed plant data, 58 of 869 (7%) of samples have had their species identification queried on the basis of their barcoding results, of which 14 (2% of all samples) have had their name changed (with 20 queries still marked as open or unresolved).

An example of how DNA barcoding has informed species identification in plants is in the weedy brassica genus *Rorippa*. A sample collected at a pond margin in the Pentland Hills, near Edinburgh, was initially identified as *Rorippa palustris*. However, two fruit traits did not match this species, casting doubt on the original identification. DNA barcoding provided a match to *R. islandica*, a congener reported from this site and also known to favour damp conditions, and matching based on these morphological traits, allowing us to confidently implement a name change. In another case, two *Geranium* accessions identified, based on morphology, as *G. endressii* and *G. versicolor* were flagged by DNA barcoding as potential hybrids, based on heterozygous positions ("wobbles") in ITS2. Photographs of both species were subsequently verified by a specialist referee, as *Geranium* x *oxonianum*, a named hybrid between *G. endressii* and *G. versicolor*, with our specimens at either end of the spectrum of variability within the hybrid. Based on this, neither of these accessions will be used for genome sequencing. However, both remain as DToL collections, with valuable metadata, field images, herbarium specimens, tissue samples, genome size data and DNA barcodes.

Beyond these groups, general issues that have arisen are that some taxa such as marine invertebrates have proven challenging for DNA extraction; some mixed samples have consistently shown non-target amplification; very small individuals have proven difficult where DNA recovery is low. With fungi, the main issue is the high levels of contamination when samples are cultured to increase biomass and pervasive cryptic diversity that challenges even the barcode identification of most common species, often revealing the existence of more than one species. To mitigate these issues GALs continue to perform R&D and protocol development. To minimise delays, taxon groups that are easy to identify based on morphology but persistently difficult to barcode, such as large mucilaginous marine molluscs, have been considered barcoding exempt (see lists in the lab SOPs). The most likely analysis issues are those groups with limited reference data, though the exact impact is hard to quantify.

## Future perspective

DNA barcoding has proved an important tool for verifying taxon identification as part of DToL. Future developments will look to further improve scalability to match the plans for more extensive genome sequencing planned for the future. Barcode focused projects such as BioScan have improved scalability by sequencing highly multiplexed amplicons, including large sample sizes sequenced with PacBio HiFi and over 100,000 samples on the ONT MinION ( Hebert *et al.*, 2023). We will investigate the utility of this approach, and others such as low-coverage genome skimming ( Hollingsworth *et al.*, 2016), for the next phase of DToL, though any move to greater genomic coverage in the barcoding phase must not compromise sample throughput and cost effectiveness. Where targeted sequencing approaches are required, the genome data generated by DToL provide an opportunity for discovering new barcode regions. Similarly, streamlining the bioinformatic analyses is a priority, and could involve the development of a unified bioinformatic engine building on the framework developed here. This next phase of DToL will also bring new challenges beyond scalability, as we move from common taxa that are easy to collect and identify, to rarer taxa, smaller species, cryptic species and those in poorly studied groups. We anticipate DNA barcoding becoming of increasing value in each phase of the project.

## Ethics and consent

Ethical approval and consent were not required.

## Data Availability

No data are associated with this article.

## References

[ref-1] Allen DE : The naturalist in Britain: a social history.London: Allen Lane,1976. Reference Source

[ref-2] Bickford D Lohman DJ Sodhi NS : Cryptic species as a window on diversity and conservation. *Trends Ecol Evol.* 2007;22(3):148–155. 10.1016/j.tree.2006.11.004 17129636

[ref-3] Crowley L Allen H Barnes I : A sampling strategy for genome sequencing the British terrestrial arthropod fauna [version 1; peer review: 2 approved]. *Wellcome Open Res.* 2023;8(123):123. 10.12688/wellcomeopenres.18925.1 37408610 PMC10318377

[ref-30] Cuber P Chooneea D Geeves C : Comparing the accuracy and efficiency of third generation sequencing technologies, Oxford Nanopore Technologies, and Pacific Biosciences, for DNA barcode sequencing applications. *Ecol Genet Genom.* 2023;28:100181. 10.1016/j.egg.2023.100181

[ref-4] Darwin Tree of Life Project Consortium: Sequence locally, think globally: The Darwin tree of life project. *Proc Natl Acad Sci U S A.* 2022;119(4): e2115642118. 10.1073/pnas.2115642118 35042805 PMC8797607

[ref-5] DeSalle R Goldstein P : Review and interpretation of trends in DNA barcoding. *Front Ecol Evol.* 2019;7:302. 10.3389/fevo.2019.00302

[ref-6] Gostel MR Kress WJ : The expanding role of DNA barcodes: indispensable tools for ecology, evolution, and conservation. *Diversity.* 2022;14(3):213. 10.3390/d14030213

[ref-7] Guillou L Bachar D Audic S : The Protist Ribosomal Reference database (PR ^2^): a catalog of unicellular eukaryote small sub-unit rRNA sequences with curated taxonomy. *Nucleic Acids Res.* 2013;41(Database issue):D597–D604. 10.1093/nar/gks1160 23193267 PMC3531120

[ref-8] Hanner R : Data standards for BARCODE records in INSDC (BRIs). 2012. 10.5479/10088/96518

[ref-9] Harding PT : Biological recording of changes in British wildlife.HMSO,1992;26. Reference Source

[ref-11] Hebert PD Floyd R Jafarpour S : Barcode 100K Specimens: In a Single Nanopore Run. *bioRxiv.* 11.29.569282,2023. 10.1101/2023.11.29.569282 PMC11646297 39387679

[ref-10] Hebert PDN Cywinska A Ball SL : Biological identifications through DNA barcodes. *Proc Biol Sci.* 2003;270(1512):313–321. 10.1098/rspb.2002.2218 12614582 PMC1691236

[ref-12] Hollingsworth PM Graham SW Little DP : Choosing and using a plant DNA barcode. *PLoS One.* 2011;6(5): e19254. 10.1371/journal.pone.0019254 21637336 PMC3102656

[ref-13] Hollingsworth PM Li DZ van der Bank M : Telling plant species apart with DNA: from barcodes to genomes. *Philos Trans R Soc Lond B Biol Sci.* 2016;371(1702): 20150338. 10.1098/rstb.2015.0338 27481790 PMC4971190

[ref-14] Jones L Twyford AD Ford CR : Barcode UK: A complete DNA barcoding resource for the flowering plants and conifers of the United Kingdom. *Mol Ecol Resour.* 2021;21(6):2050–2062. 10.1111/1755-0998.13388 33749162

[ref-15] Kress WJ García-Robledo C Uriarte M : DNA barcodes for ecology, evolution, and conservation. *Trends Ecol Evol.* 2015;30(1):25–35. 10.1016/j.tree.2014.10.008 25468359

[ref-16] Lawniczak MK Davey RP Rajan J : Specimen and sample metadata standards for biodiversity genomics: a proposal from the Darwin Tree of Life project [version 1; peer review: 2 approved with reservations]. *Wellcome Open Res.* 2022a;7(187):187. 10.12688/wellcomeopenres.17605.1 PMC11292180 39091415

[ref-17] Lawniczak MK Durbin R Flicek P : Standards recommendations for the earth BioGenome project. *Proc Natl Acad Sci U S A.* 2022b;119(4): e2115639118. 10.1073/pnas.2115639118 35042802 PMC8795494

[ref-18] Lücking R Aime MC Robbertse B : Unambiguous identification of fungi: where do we stand and how accurate and precise is fungal DNA barcoding? *IMA Fungus.* 2020;11(1): 14. 10.1186/s43008-020-00033-z 32714773 PMC7353689

[ref-19] Martay B Brewer MJ Elston DA : Impacts of climate change on national biodiversity population trends. *Ecography.* 2017;40(10):1139–1151. 10.1111/ecog.02411

[ref-20] Nilsson RH Larsson KH Taylor AFS : The UNITE database for molecular identification of fungi: handling dark taxa and parallel taxonomic classifications. *Nucleic Acids Res.* 2019;47(D1):D259–D264. 10.1093/nar/gky1022 30371820 PMC6324048

[ref-21] Padial JM Miralles A De la Riva I : The integrative future of taxonomy. *Front Zool.* 2010;7(1): 16. 10.1186/1742-9994-7-16 20500846 PMC2890416

[ref-22] Pereira L Sivell O Sivess L : DToL Taxon-specific Standard Operating Procedure for the Terrestrial and Freshwater Arthropods Working Group. 2022. 10.17504/protocols.io.261gennyog47/v1

[ref-23] Pocock MJ Roy HE Preston CD : The Biological Records Centre: a pioneer of citizen science. *Biol J Linn Soc.* 2015;115(3):475–493. 10.1111/bij.12548

[ref-24] Price BW Briscoe AG Misra R : Evaluation of DNA barcode libraries used in the UK and developing an action plan to fill priority gaps. *Nat Engl.* 2020.

[ref-25] Quast C Pruesse E Yilmaz P : The SILVA ribosomal RNA gene database project: improved data processing and web-based tools. *Nucleic Acids Res.* 2012;41(Database issue):D590–D596. 10.1093/nar/gks1219 23193283 PMC3531112

[ref-26] Ratnasingham S Hebert PDN : bold: The Barcode of Life Data System ( http://www.barcodinglife.org). *Mol Ecol Notes.* 2007;7(3):355–364. 10.1111/j.1471-8286.2007.01678.x 18784790 PMC1890991

[ref-27] Ratnasingham S Hebert PDN : A DNA-based registry for all animal species: the barcode index number (BIN) system. *PLoS One.* 2013;8(7): e66213. 10.1371/journal.pone.0066213 23861743 PMC3704603

[ref-28] Stroh PA Walker KJ Humphrey TA : Plant atlas 2020: mapping changes in the distribution of the British and Irish flora.Princeton University Press,2023. 10.2307/j.ctv2x6f08m

[ref-29] Threlfall J Blaxter M : Launching the Tree of Life Gateway [version 1; peer review: not peer reviewed]. *Wellcome Open Res.* 2021;6:125. 10.12688/wellcomeopenres.16913.1 34095514 PMC8142598

