# Supplementary material for: A DNA barcoding framework for taxonomic verification in the Darwin Tree of Life Project
Source: Wellcome Open Res. 2024 Jun 24;9:339. [Version 1] doi: 10.12688/wellcomeopenres.21143.1 (PMC11462125; doi:10.12688/wellcomeopenres.21143.1)
Supplement: Peer review history

# Reviewer report: Reviewer response for version 1

**DOI:** 10.21956/wellcomeopenres.23387.r93648

**Published:** 2024-10-08

**Authors:** Georgi Bonchev

**Affiliations:** 1 Bulgarian Academy of Sciences, Sofia, Bulgaria

**Summary of the article**

 The authors present the Darwin Tree of Life Project (DToL) project aimed at sequencing and assembling high quality reference genomes for all eukaryotic species present in Britain and Ireland. The paper presents a framework of comprehensive taxonomic double-check characterization of samples based on both DNA barcoding and morphological characterization. The outcomes serve as a platform for development of standardized protocols and decision making how to deal with them and that can be applied on a large scale across different taxonomic groups and laboratories.

 The project has an important value not only for the contribution to the enrichment of UK and Ireland databases but this approach can be also adopted by other countries in their large-scale biodiversity genome initiatives.  

 Therefore, I suggest the manuscript as relevant for Indexing.

 Here below I will provide some comments/questions/suggestions to authors that may make the manuscript clearer and more informative for readers.

**Comments**

 1. Is a blind approach used when comparing DNA barcoding and morphological data? This may be mentioned.

 2. Morphological identification

 It seems that morphological identification cannot be comprehensively performed in the field at the collection sites and requires lab analyses. Perhaps, the authors should revise a bit the sentence “Initially, all specimens are identified in the field at the point of collection by taxonomic experts" or to extend it that this task continues further in labs. It is explained in more details in the beginning of section *Embedding DNA barcoding in the sample workflow*

**3. Citation: “Within BOLD, for example, at least one barcode sequence was available for 42.5% of UK species, however in many cases this was not from a specimen collected from the UK”**

 I do not quite understand how barcodes for UK species are collected from specimens not from UK? You mean specimens from other collections worldwide or……… :

**4.** The Figure 1 Include not only the workflow of DNA barcoding but also species verification by taxonomists at the later stage. Therefore, the title should be more general DToL species identification workflow (not only DNA barcoding) or similar. It seems reasonable to depict in Figure 1 other match categories and outcomes from DNA barcoding queries (not only the definitive match) and which lead to genome sequencing as mentioned in Table 3. The Fig. 1 shows that specimens with >99% match go directly to BOLD without taxonomic expertise. These samples obviously also undergo taxonomic expertise and this may be shown.

 The authors talk about DTooL DNA barcoding hubs but also mention DToL taxon working groups. Are these one and the same or the latter ones are upper levels of coordination and species identification (comprising molecular biologists and classical taxonomists)?

 Exemption lists

**5. The workflow in practice**

**Citation: “The BOLD query produced a match with the expected species in 59.2% of cases, eliminating the need for further checks” **Do the taxonomic affiliation of these samples are not confirmed by taxonomists?

**It seems relevant the information for animals for different outcomes of identifications or failures to be presented for other main taxa – plants, fungi:**

 - Number of samples that had their change from identifier ID to BOLD ID

 - It is interesting to know the fraction of species for which DNA barcoding identification fails comparing to morphological identification (examples)

 - Number of new species described due to DNA barcoding (either due to reevaluation of known previously identified species or samples not previously taxonomically characterized or new for the science) - examples

 As the DToL addresses the goal to sequence high quality reference genomes, it seems reasonable authors to show how many genomes (from which genus, species) were sequenced in the course of the project for different taxa.

 What criteria was chosen for sequencing: only the criteria in Table 3 (1, 2 and 4) as this is a huge number of species or there were other selections criteria?

Does the article adequately reference differing views and opinions?

Yes

Are all factual statements correct, and are statements and arguments made adequately supported by citations?

Yes

Is the Open Letter written in accessible language?

Yes

Where applicable, are recommendations and next steps explained clearly for others to follow?

Yes

Is the rationale for the Open Letter provided in sufficient detail?

Yes

Reviewer Expertise:

plant molecular biology, DNA molecular markers, DNA barcoding for biodiversity, evolutionary studies, abiotic stress

I confirm that I have read this submission and believe that I have an appropriate level of expertise to confirm that it is of an acceptable scientific standard.

# Reviewer report: Reviewer response for version 1

**DOI:** 10.21956/wellcomeopenres.23387.r96790

**Published:** 2024-09-20

**Authors:** Gilles San Martin

**Affiliations:** 1 Walloon Agricultural Research Centre, Gembloux, Belgium

**Summary :**

 The Darwin Tree of Life Project (DToL) aims to sequence the complete genomes of all eukaryotes from the UK and Ireland. Specimens are identified by experts, but identification errors are always possible and attributing a full genome sequence to the wrong species would be problematic.

 The authors propose to double-check expert identifications with barcoding and to further investigate identification mismatches (which may have several explanations) in consultation with these experts. There is a need for a standardized protocol and decision process that can be applied on a large scale across different taxonomic groups and laboratories. An interesting side product of this approach is also to enrich the existing barcoding databases with new sequences and high quality metadata.

**General comment :**

 I don't see any major problems with the proposed approach and I don't see any reason to withhold publication.

 In my opinion, the proposed approach is very robust in that the identifications are not based on barcoding alone, but compare both the identifications made by taxonomy experts and those made through barcoding. In cases of disagreement, which is right or wrong and why, needs to be investigated and agreed between the molecular biologists in the lab and the field experts in order to improve the level of confidence in the final identification.

 A blind spot could be that even with this double check, identification errors and future taxonomic changes are still likely. The only protection against this problem would be

 1) to have high quality associated data/metadata (including museum specimens and high quality images including morphological details needed for identification) 

 2) a protocol for receiving feedback (e.g. if someone suspects an identification error) and handling taxon name changes in the future 

 3) a way to document these changes with explanations/justifications.

 But this is maybe beyond the scope of this paper.

 I have a lot of little (and not so little) comments and questions, but I don't think it's necessary to answer all of them (the authors should have the choice to ignore these comments or not).

**Two related more general comments :**

 1)  Exemption lists

 "Exemption lists" are mentioned only twice in the paper (if I'm not mistaken) but there is very little details. SOPs are mentioned but do not seem to be accessible (?) and I'm not certain to understand the exact use cases for these lists.

 It looks like these lists are only for groups for which DNA extraction or amplification is problematic but not (?) for taxonomic groups for which the barcodes are known to be either misleading or not useful because they do not allow the discrimination between closely related species. Wouldn't be interesting to also have lists for these second cases too?

 Citations from the paper :

 " Finally, as there are a small number of taxa where morphological identification is certain, but barcode sequencing is known to have very low success, we have instituted a barcoding exemption list. (...) (discussed more below)"  (...)

 "To minimise delays, taxon groups that are easy to identify based on morphology but persistently difficult to barcode, such as large mucilaginous marine molluscs, have been considered barcoding exempt (see lists in the lab SOPs)"

 2) Flowchart to illustrate the whole decision process from the exemption lists to the final matching categorization?

 The paper describes several important modules, but it is not entirely clear to me how they articulate with each other (and within them) during the decision process:

- Exemption lists
- Taxonomic assignment strategies: 4 described (but how is the decision made to use one of them?): a "closest match", b "BOLD BIN", c "user specified threshold", d "phylogenetic approach".
- Match type classification (detailed in Table 3)

For example, the taxonomic coverage of the reference database seems to be important both in deciding which taxonomic assignment method to use and in the match type classification. So I guess this step needs to be done before both taxonomic assignment and match type classification.

 I don't know if this exists or is possible, but I would love to see a detailed decision protocol/flowchart, as if I were a new member of the lab and had to make all the decisions myself. I understand that this is the kind of detailed information you would put in an SOP rather than a scientific paper. But are such SOPs publicly available? Would it be possible to make a reasonably sized decision flowchart for the paper? Perhaps just as an example for one taxonomic group?

**Minor comments:**

 3) Figure 1

- IMHO, Fig. 1 is not very interesting and might even be misleading : the first extraction/amplification/sequencing steps are obvious, and the last part of the flowchart looks like the only criterion used is a 99% sequence identity match, while the rest of the paper (e.g. Table 3) shows that a much more careful approach is used in the decisions
-  I would prefer something a bit more detailed that better describes your decision workflow (see previous comment).  (NB : just a personal preference/opinion, not a "request"...)

4) Morphological/field identifications

 It is unclear to me how morphological identification is performed and documented. The text contains sentences like " Initially, all specimens are identified in the field at the point of collection by taxonomic experts". It looks like identification is done in the field, which is impossible for many taxa. I assume that taxonomic experts use all necessary methods for identification (including dissection of genitalia, microscopic preparation, ... if necessary). By "identifications made on the field" do you mean "morphological identifications"? This also raises questions about how specimens are handled when destructive approaches are needed (such as microscopic slide preparation for some arthropods, e.g. Coccoidea, Aphidoidea), but perhaps this is a bit outside the scope of this paper.

 5) Section "The workflow in practice"

- For Animalia, the text explains that ~60% of the barcode-based identifications matched the morphological ones, and that ~20% required additional verification (further subdivided in the text into various subcategories that add up to about 20%, if I understand correctly).
- But what about the remaining 20%?
- No detailed numbers are given for other taxonomic groups (plants, fungi, ...). I suspect, for example, that the % concordance between barcode and morphological identification is more problematic for these groups for various reasons (e.g. for plants, species-level identification is often not very good with ITS2 and even more so with rbcL).
- I would be very interested to see, for example, a table with the different cases and subcases listed for animals, but for several taxonomic groups.
- The text lists several interesting success stories where barcoding has been able to resolve problems in morphological identification. But aren't there examples where the barcoding consistently pointed to a taxon and it was ultimately decided that the morphological identification was correct?
- I'm surprised, for example, not to see cases where identification errors in the reference databases are likely to have caused the discrepancy. Or cases where the barcode is unreliable for distinguishing closely related species.

6) Taxonomic coverage of the databases

 Taxonomic coverage of the database is an important criterion to evaluate the pertinence of the barcoding taxonomic assignment outputs. But which biogeographic range is considered. Only UK and Ireland ? Larger ?

 7) User defined thresholds

 For taxonomic assignment,  option c ("user defined thresholds") seems to be preferred when databases taxonomic coverage is low. But how the thresholds are determined ? It seems difficult to evaluate which threshold would be reliable when you have few species in the database. Extrapolation from other species of the same group ?

 8) Intra-specific polymorphism in table 3 (Inconsistent match case (a) )

 I'm not sure I understand how "previously undetected intra-specific variation in the barcode region (polymorphsim)" could lead to a "match to a different but closely related species".

 Polymorphism in the barcode region could be due to :

- Identification errors in the reference database (you actually have two different species already described and there is no real polymorphism) --> you may have a mismatch, but the problem is the error in the database, not the polymorphism per se.
- Hidden taxonomic diversity: the described species is really 2 different undescribed species, but you cannot conclude this with just one barcode region --> in this case the morphological identification should match the barcode one, or you should have a "consistent match" (no satisfactory match at species level).
-  True polymorphism (true intra-specific variation), well represented in the database --> this should also lead to a "consistent match", at least in the worst case (lack of resolution).

I can maybe imagine a case where you would have intraspecific polymorphism AND lack of separation between species AND lack of coverage in the database so that the lack of separation is undetectable?

Does the article adequately reference differing views and opinions?

Yes

Are all factual statements correct, and are statements and arguments made adequately supported by citations?

Yes

Is the Open Letter written in accessible language?

Yes

Where applicable, are recommendations and next steps explained clearly for others to follow?

Yes

Is the rationale for the Open Letter provided in sufficient detail?

Yes

Reviewer Expertise:

Applied entomology, Applied data science and bioinformatics, Environment, ...

I confirm that I have read this submission and believe that I have an appropriate level of expertise to confirm that it is of an acceptable scientific standard.
